# School bullying and depression in Chinese primary and secondary school students after the COVID-19: mediating effect of academic anxiety and moderating effect of home-school cooperation

**DOI:** 10.3389/fpsyg.2025.1535067

**Published:** 2025-07-23

**Authors:** Ping Lan, Ling Lan, Wei-Ping Zhang

**Affiliations:** ^1^Library of Sichuan Minzu College, Kangding, China; ^2^School of Economics and Management of Sichuan Minzu College, Kangding, China; ^3^School of Education of Hunan University of Science and Technology, Xiangtan, China

**Keywords:** school bullying, depression, academic anxiety, home-school cooperation, primary and secondary school students

## Abstract

**Introduction:**

The aim of this study is to explore the relationship between school bullying, depression, academic anxiety, and home-school cooperation among Chinese primary and secondary school students, with a focus on promoting students' mental wellbeing in the post-COVID-19 era.

**Methods:**

A survey was conducted among 3,600 Chinese primary and secondary school students using the Depression Scale, School Bullying Scale, Academic Anxiety Scale, and Home-School Cooperation Scale. A total of 3,341 students were selected as research subjects to examine the mediating and moderating mechanisms of academic anxiety and home-school cooperation in the relationship between school bullying and depression. A moderated mediation model was constructed to analyze the impact of school bullying on depression, with academic anxiety as a mediator and home-school cooperation as a moderator.

**Results:**

School bullying, depression, and academic anxiety were significantly correlated with home-school cooperation. School bullying was significantly correlated with academic anxiety and depression. School bullying significantly and positively predicted depression. Academic anxiety mediated the relationship between school bullying and depression. Home-school cooperation moderated the relationships between (a) school bullying and academic anxiety, and (b) academic anxiety and depression.

**Discussion:**

The findings indicate that school bullying not only directly increased depression but also indirectly heightened depression through academic anxiety. Home-school cooperation buffered the effects of school bullying on academic anxiety and the effects of academic anxiety on depression.

## 1 Introduction

Depression has been an unneglectful psychological problem among Chinese primary and secondary school students for a long time. Depression is a kind of negative emotion produced by internal and external environment, which is manifested as distress, irritability, sadness, unpleasantness, continuous depression and so on (Xiao et al., 2023). After the outbreak of COVID-19, the depression detection rate of primary and secondary school students in the world reached 25.2% (Zhu et al., 2021), and the depression detection rate of Chinese primary and secondary school students reached 43.7% (Zhou et al., 2020). Primary and secondary school students are in a period of continuous psychological and physical development, they tend to be emotionally unstable, after the epidemic, primary and secondary school students are more prone to depression and other negative emotions. A mild degree of depression has a negative impact on the normal study and life of primary and secondary school students, while a serious degree of depression may even lead to self-harm, suicide and other self-harming behaviors (Guo and Zhang, 2003).

In recent years, students' physical and mental healthy growth has been paid more and more attention in China. Students' physical and mental healthy growth includes both physical and mental healthy growth. Depression is one of the important psychological problems that hinder students' mental healthy growth, depression and its developmental patterns among primary and secondary school students have always been an important area of emotional research (Liu, 1997). Exploring the antecedents and mechanisms of depression among primary and secondary school students is helpful to provide a basis for the prevention and intervention of depression in theory and practice. Terefore, there is a need to understand the risk factors and potential mechanisms of depression to better prevent and treat depression in Chinese primary and secondary school students after the COVID-19.

The adaptive load theory suggests that the overload operation of an individual's adaptive system during stress can disrupt its function (Mcewen, 2010). School bullying is a special kind of aggressive behavior (Awiria et al., 1994), victims of school bullying are intentionally, repeatedly, and consistently subjected to negative behavior by one or more students, causing physical and psychological harm or maladjustment (Olweus, 1994). Experiencing school bullying as a chronic stressor that can disrupt an individual's hypothalamic pituitary adrenal axis stress function can increase the risk of developing emotional disorders or psychological disorders (Kong and Chen, 2017). If primary and secondary school students who have been bullied are in this stress state for a long time, it is highly likely to trigger adverse mental health problems such as depression (Mcewen, 2010). The types of school bullying mainly include relationship bullying, verbal bullying, physical bullying, cyber bullying, etc (Hu and Li, 2018). School bullying is an important factor affecting students' mental health (Jadambaa et al., 2019). No matter what form of school bullying, being in it for a long time is likely to force these students bullied to withdraw from the mainstream social group and be marginalized in the peer group, which will lead to internalized and externalized psychological problems (Ji et al., 2011). Bullied students are prone to behaviors such as anxiety, depression and even suicidality (Lv et al., 2022; Yu et al., 2020). Students who experience school bullying are at higher risk for mental health problems such as depression, anxiety, attention deficit hyperactivity disorder, and conduct disorders (Kretschmer, 2017), and depression is the most strongly associated with school bullying (Chen et al., 2020). A survey on primary and secondary school students in China showed that the risk of anxiety and depression among bullied students is 3.96 times (Gong et al., 2022). According to the research by Zhao Jingxin, Yang Ping, Zhao Xijia and Zhang Wenxin, school bullying is an important factor in depression (Zhao et al., 2016), the research by Chen Qi-Qi, Chen Meng-Tong and Zhu Yu-Hong also discovered that students who experience school bullying are more likely to suffer from depression (Chen et al., 2018). Victims of school bullying may develop depression immediately after the bullying occurs, or they may develop depression after a period of time following the bullying (Stapinski et al., 2015). Sometimes, the effects of school bullying victimization on depression persist into adulthood (Copeland et al., 2013).

Depression among primary and secondary school students is closely related to bullying behavior. Students who have been bullied for a long time may experience depression symptoms (Zhao et al., 2024). However, previous studies mainly focus on the direct impact of school bullying on depression, and pay insufficient attention to the potential mediating effects and moderating mechanisms between school bullying and depression. Terefore, this study proposed a moderated mediation model to explain the intrinsic mechanisms between school bullying and depression.In this study, academic anxiety is used as a mediating variable, and home-school cooperation is used as a moderating variable, examining the direct and indirect effects of school bullying on depression.

### 1.1 Mediating effect of academic anxiety

Academic anxiety, as one of the main mental health problems faced by primary and secondary school students, refers to the negative mental state experienced by students in the academic situation (Pekrun et al., 2002). Academic anxiety is mainly manifested as tension, unease, fear, and apprehension toward learning tasks and processes (Dong and Yu, 2010), Huang Liang and Zhao Decheng discovered that being bullied at school can increase students' academic anxiety (Huang and Zhao, 2020). Students who are bullied in school are often in a state of fear, and they are prone to feeling nervous and uneasy. In the meantime, they are unable to concentrate in class and their academic performance continues to decline, leading to severe academic anxiety (Zhang and Chen, 2016). School bullying will damage students' learning motivation, academic self-confidence, seriously restrict students' academic participation level and learning effect, trigger students' social withdrawal behavior, and then cause students to have academic anxiety (Horton, 2011). Compared with students who have not been bullied in school, students who have been bullied in school are more likely to have academic anxiety, and students with academic anxiety may hold negative self-belief and cowardice. Long-term severe academic anxiety will gradually lead students to accept unsatisfactory results, and then lead to their depression and other negative emotions (Sun and Chen, 2010). As school bullying has a strong direct impact on academic anxiety, and academic anxiety is a predictor of depression, this suggests that academic anxiety has a mediating effect. Terefore, this study hypothesizes that academic anxiety may have a mediating effect on the relationship between school bullying and depression.

### 1.2 Moderating effects of home-school cooperation

Home-school cooperation is an interactive educational activity, which requires family and school to cooperate, support and coordinate with each other for the purpose of promoting students' development (Liu et al., 2007). Family and school are important factors affecting school bullying, academic anxiety and depression (Chen et al., 2009). The family element of school bullying is often manifested in the family's inability to develop children's basic survival skills and the lack of caring needed for children's mental health development (Su et al., 2016). Sometimes, parents ignore children's social interactions because they are busy with work or only focus on their children's academic performance. As a result, many bullied students do not report the harm they have suffered to their parents or teachers. Teachers do not fully understand the frequency and severity of bullying behavior, and they also downplay it for fear of being accused of inadequate supervision or being detrimental to the school, making it difficult to effectively intervene in bullying incidents (Hu, 2017). Parents' psychological control, such as ridicule, invasion of privacy, and excessively high expectations, is significantly positively correlated with children's academic anxiety (Li and Gu, 2023). Psychological assistance and effective intervention from schools can alleviate students' academic anxiety (Liu, 2009). Family can have an impact on adolescent depression in many ways. Parents who adopt negative parenting styles and lack positive emotional support may increase the likelihood of adolescent depression (Yi and Qian, 2006). In the context of campus culture, the most important people for teenagers are teachers and classmates. If teenagers feel more interpersonal support, their risk of depression will be reduced (Xu et al., 2014). Home-school cooperation communicates the two worlds of students' families and schools (Huang and Ma, 2011). Good home-school cooperation can help prevent teenagers from making mistakes, make up for some deficiencies in education when preventive education fails, and help teenagers develop physically and mentally in accordance with the requirements of the national education policy (Chen, 2018). Considering that home-school cooperation can alleviate the adverse effects of life pressure on students' mental health, it can be inferred that students with high level home-school cooperation have better mental ability to adapt to and bear the pressure brought by life when facing school bullying. Terefore, home-school cooperation can alleviate the effects of school bullying on academic anxiety and the effects of academic anxiety on depression. This means that home-school cooperation has moderating effects on the relationship between school bullying and academic anxiety and the relationship between academic anxiety and depression.

### 1.3 The present study

**Figure 1:**
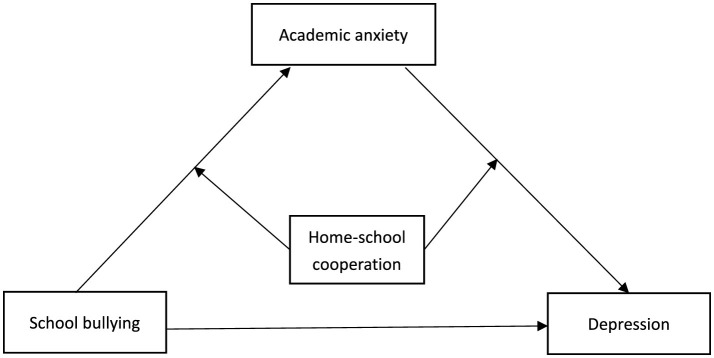
The proposed moderated mediation model.

Many education policies in China have always explicitly stated the need to pay sufficient attention to students' physical and mental health. Depression is one of the important psychological problems that hinder students' mental health (Ren and Tang, 2014). With the rapid economic and social development, students' growth environment is constantly changing, combined with the impact of the COVID-19 epidemic, students' mental health problems are more prominent (Troop and Ladd, 2005). The objective of this study is to examine the relationship between school bullying and depression in Chinese primary and secondary school students after the COVID-19 and further examine the mediating and moderating mechanisms involved to construct a moderated mediation model (as shown in Figure 1). Therefore, we proposed 3 study hypotheses: (1) School bullying is a signifcant positive predictor of depression. (2) Academic anxiety has a mediating effect on the relationship between school bullying and depression. (3) Home-school cooperation has moderating effects on the relationship between school bullying and academic anxiety and the relationship between academic anxiety and depression.

## 2 Methods

### 2.1 Participants and processes

This study was approved by the Ethics Committee of the school, and consent was obtained from teachers and parents, and informed consent forms were signed with the students themselves. Before starting the questionnaire survey, we provided training to the homeroom teachers who assisted in the survey. During the investigation process, the homeroom teacher explained the significance of the survey, emphasized the confidentiality of the questionnaire content, and guided students to maintain an appropriate distance when filling out the questionnaire. After the investigation is completed, the homeroom teacher verifies the submitted questionnaire. Convenience sampling was employed, and students from the first grade of primary school to the third grade of high school of six provinces in China, including Hubei, Hunan, Shandong, Guangdong, Sichuan, and Guangxi were recruited as study subjects. Each homeroom teacher distributed questionnaires in class and asked students to complete the questionnaire within 15 minutes. The time for collecting the questionnaire was from November 13th to 17th, 2023. A total of 3,600 questionnaires were collected, and 3,341 valid questionnaires were finally sorted out by removing outliers through the Mahalanobis distance, resulting in a final valid response rate of 92.81%.

### 2.2 Measurements

#### 2.2.1 Demographic variables

Demographic variables, such as gender, grade, and province were controlled in the subsequent analysis. Specifcally, the variables included gender (0 = female, 1 = male), grade (1 = second grade, 2 = third grade, 3 = fourth grade, 4 = fifth grade, 5 = sixth grade, 6 = seventh grade, 7 = eighth grade, 8 = ninth grade, 9 = tenth grade, 10 = eleventh grade, 11 = twelfth grade), and province (1 = Hubei, 2 = Hunan, 3 = Shandong, 4 = Guangdong, 5 = Sichuan, 6 = Guangxi). We divided the scores of the school bullying scale, depression scale, academic anxiety scale, and home-school cooperation scale into three levels: low, medium, and high. For the school bullying scale, scores ≥0 but < 7 are considered “low,” scores ≥7 but < 14 are considered “medium,” and scores ≥14 but < 21 are considered “high.” For depression scales, scores ≥0 and < 8 are considered “low,” scores ≥8 and < 16 are considered “medium,” and scores ≥16 and < 24 are considered “high.” For the academic anxiety scale, scores ≥0 and < 5 are considered “low,” scores ≥5 and < 10 are considered “medium,” and scores ≥10 and < 15 are considered “high.” For the home-school cooperation scale, scores ≥0 and < 6 are considered “low,” scores ≥6 and < 12 are considered “medium,” and scores ≥12 and < 18 are considered “high.”

#### 2.2.2 School bullying

The school bullying scale of this study drew on the six items of the PISA 2018 school bullying scale, and added one item based on the research of Hu Yongmei and Li Jiazhe (Hu and Li, 2018), for a total of seven items to measure the situation of primary and secondary school students suffering from school bullying. The content of the school bullying scale in this study includes “the number of times other students intentionally isolate and exclude me; the number of times other students mock and ridicule me; the number of times I am threatened by other students; the number of times other students intentionally take away or damage my property; the number of times I am hit and pushed by other students; the number of times other students spread rumors about me; the number of times I am subjected to verbal attacks, information harassment, insulting pictures or videos from others on the internet.” The school bullying scale items are scored from 0 to 3 points (0 = never; 1 = multiple times a year; 2 = multiple times a month; 3 = once a week or more), with a total score of 0–21 points. The higher the score of the respondents, the more severe the degree of school bullying they have experienced. The Cronbach's Alpha coefficient of the school bullying scale in this study was 0.75 (0.75 > 0.7), indicating high reliability and good internal consistency of the scale. The KMO value for validity analysis of the school bullying scale was 0.82 (0.82 > 0.6), with *p* = 0.000 < 0.05, indicating high validity of the scale.

#### 2.2.3 Academic anxiety

The academic anxiety scale used in this study was developed based on the China Education Panel Studies (CEPS) questionnaire and consists of five questions. The content of the academic anxiety scale used in this study is “I often worry that exams are too difficult; I worry that I will score very low in the exam; I feel very nervous when reviewing and preparing for exams; even if I have made sufficient preparations before exams, I am still very anxious; I feel very nervous when encountering problems that cannot be solved in school.” The academic anxiety scale used in this study adopted the Likert four-point rating system, where 0 = strongly disagree, 1 = disagree, 2 = agree, and 3 = strongly agree. The total score ranged from 0 to 15 points, and the higher the score of the respondent, the more severe the academic anxiety. The Cronbach's Alpha coefficient of the academic anxiety scale in this study was 0.90 (0.90 > 0.7), indicating high reliability and good internal consistency of the scale. The KMO value of validity analysis was 0.85 (0.85 > 0.6), with *p* = 0.000 < 0.05, indicating high validity of the scale.

#### 2.2.4 Home-school cooperation

The home-school cooperation scale used in this study was developed based on the China Education Panel Survey (CEPS) questionnaire and consists of six items, including “the number of times your parents attend parent meetings; the number of times your teachers report your situation at school to your parents; the number of times your teachers invite your parents to attend classes at school; the number of times your school invites your parents to attend teacher symposiums; the number of times your teachers invite your parents to watch performances or participate in extracurricular activities organized by the school; the number of times your school hold parent-child lectures.” The home-school cooperation scale adopted the Likert four-point rating system, with 0 = none, 1 = once, 2 = two to four times, and 3 = five or more times. The total score ranges from 0 to 18 points, and the higher the score of the respondent, the better the cooperation between home and school. The Cronbach's Alpha coefficient of the home-school cooperation scale was 0.84 (0.84 > 0.7), indicating high reliability and good internal consistency. The KMO value of validity analysis was 0.87 (0.87 > 0.6), with *p* = 0.000 < 0.05, indicating high validity of the scale.

#### 2.2.5 Depression

The depression scale selected in this study was mainly developed by Ren Qiang and Tang Qiming (Ren and Tang, 2014). This depression scale is widely used in the study of depression and can effectively measure students' depressive symptoms (Troop and Ladd, 2005). The depression scale of this study consists of six items, and for each item, respondents need to answer their own feelings—“almost every day,” “two or three times a week,” “two or three times a month,” “once a month,” “never.” Each option is assigned a score of 0 (“never”) to 4 (“almost every day”), with a total score of 0–24. The higher the respondent's score, the more severe their depression. In this study, the Cronbach's Alpha coefficient of the scale was 0.86 (0.86>0.7), indicating high reliability and good internal consistency of the scale. The KMO value for validity analysis was 0.82 (0.82 > 0.6), with *p* = 0.000 < 0.05, indicating high validity of the scale.

### 2.3 Data analysis

All data analysis was performed using SPSS 26.0. A value of p of 0.05 indicated statistical significance. First, frequency (percentage) or mean (standard deviation) were used to calculate demographic variables, including gender, grade, and province. Independent *t*-test, One-way ANOVA or Pearson' s correlation analysis were used to examine the relationship between these demographic variables and school bullying, depression, academic anxiety, and home-school cooperation based on the study objective and data type. Second, we used the SPSS PROCESS macro (model 4 and model 58) proposed by Hayes (Hayes, 2013) to validate the moderated mediation model. A bootstrapping procedure was selected with 5,000 bootstrap samples used to calculate bias corrected 95% confidence intervals. When the confidence interval of the model parameters does not include 0, the results are statistically significant. In addition, all potential significant interactions were analyzed using simple gradients (Toothaker, 1994).

## 3 Results

### 3.1 Descriptive statistics

The results of descriptive statistics are shown in Tables 1, 2. Table 1 shows that the grade range of the subjects was from second grade of primary school to senior grade three, of which 34% were elementary school students, 39.87% were middle school students, and 26.13% were high school students. Among these participants, 1,680 (50.28%) were male and 1,661 (49.72%) were female, and 567 from Hubei, 497 from Hunan, 645 from Shandong, 730 from Guangdong, 620 from Sichuan, 282 from Guangxi. Table 2 shows participants' scores of the school bullying scale, depression scale, academic anxiety scale, and home-school cooperation scale. From the scores of the participants, it was evident that male students suffered more from school bullying than female students, while female students had higher levels of depression. Most of the 3,341 students had higher levels of academic anxiety, and there were fewer students with higher levels of home-school cooperation.

**Table 1:** Participants' sociodemographic characteristics.

**Variables**	**Category**	**Frequency**	**Percentage (%)**	**Mean value**	**Standard deviation**
Province	Hubei	567	16.97	3.35	1.56
	Hunan	497	14.88		
	Shandong	645	19.31		
	Guangdong	730	21.85		
	Sichuan	620	18.56		
	Guangxi	282	8.44		
Gender	Females	1,661	49.72		
	Males	1,680	50.28		
Grade	Second grade	1	0.03	7.61	2.04
	Third grade	21	0.63		
	Fourth grade	62	1.86		
	Fifth grade	581	17.39		
	Sixth grade	471	14.10		
	Seventh grade	487	14.58		
	Eighth grade	686	20.53		
	Ninth grade	159	4.76		
	Tenth grade	562	16.82		
	Eleventh grade	301	9.01		
	Twelfth grade	10	0.30		

**Table 2:** Participants' score of each scale *n* (%).

**Scale**	**School bullying**			**Depression**			**Academic anxiety**			**Home-school cooperation**		
Score	0–7	7–14	14–21	0–8	8–16	16–24	0–5	5–10	10–15	0–6	6–12	12–18
Males	1,607 (48.10)	59 (1.77)	14 (0.42)	1,194 (35.74)	387 (11.58)	99 (2.96)	414 (12.39)	691 (20.68)	575 (17.21)	851 (25.47)	553 (16.55)	276 (8.26)
Females	1,611 (48.22)	41 (1.23)	9 (0.03)	1,065 (31.88)	446 (13.35)	150 (4.45)	272 (8.14)	711 (21.28)	678 (20.29)	759 (22.72)	610 (18.26)	292 (8.74)
Primary school students	1,086 (32.51)	37 (1.11)	13 (0.39)	905 (27.09)	178 (5.33)	53 (1.59)	384 (11.49)	453 (13.56)	299 (8.95)	394 (11.79)	452 (13.53)	290 (8.68)
Middle school students	1,284 (38.43)	43 (1.29)	5 (0.15)	849 (25.41)	369 (11.05)	114 (3.41)	195 (5.84)	539 (16.13)	598 (17.90)	620 (18.56)	496 (14.85)	216 (6.47)
High school students	848 (25.38)	20 (0.60)	5 (0.15)	505 (15.12)	286 (8.56)	82 (2.45)	107 (3.20)	410 (12.27)	356 (10.66)	596 (17.84)	215 (6.44)	62 (1.86)
Students from Hubei	551 (16.49)	14 (0.42)	2 (0.06)	389 (11.64)	138 (4.13)	40 (1.20)	100 (2.99)	241 (7.21)	226 (6.76)	369 (11.05)	161 (4.82)	37 (1.11)
Students from Hunan	471 (14.10)	23 (0.69)	3 (0.09)	329 (9.85)	122 (3.65)	46 (1.38)	98 (2.93)	195 (5.84)	204 (6.11)	210 (6.29)	197 (5.90)	90 (2.69)
Students from Shandong	633 (18.95)	10 (0.30)	2 (0.06)	489 (14.64)	130 (3.89)	26 (0.78)	206 (6.17)	249 (7.45)	190 (5.69)	254 (7.60)	178 (5.33)	213 (6.38)
Students from Guangdong	691 (20.68)	26 (0.78)	13 (0.39)	420 (12.57)	226 (6.76)	84 (2.51)	86 (2.57)	323 (9.67)	321 (9.61)	431 (12.90)	247 (7.39)	52 (1.56)
Students from Sichuan	596 (17.84)	21 (0.63)	3 (0.09)	424 (12.69)	163 (4.88)	33 (0.99)	147 (4.40)	246 (7.36)	227 (6.79)	228 (6.82)	277 (8.29)	115 (3.44)
Students from Guangxi	276 (8.26)	6 (0.18)	0 (0.00)	208 (6.23)	54 (1.62)	20 (0.60)	49 (1.47)	148 (4.43)	85 (2.54)	118 (3.53)	103 (3.08)	61 (1.83)

### 3.2 Comparative analysis

Table 3 yields several critical findings. There were significant differences in school bullying, depression, and academic anxiety between male and female primary and secondary school students (*p* < 0.001), as well as significant differences in home-school cooperation (*p* < 0.05). Specifically, male students had a higher level of school bullying than female students, female students had higher levels of depression and academic anxiety, and female students had slightly higher levels of home school cooperation than male students. There were significant differences (*p* < 0.001) in school bullying, depression, academic anxiety, and home-school cooperation among primary and secondary school students of different grades. In terms of school bullying, the degree of school bullying was slightly higher among primary school students and middle school students than among high school students. In terms of depression, middle school students and high school students had higher levels of depression than primary school students. In terms of academic anxiety, almost all students who participated in the questionnaire survey of this study generally had a certain degree of academic anxiety, with middle school students and high school students having higher levels of academic anxiety than primary school students. In terms of home-school cooperation, the degree of home-school cooperation was relatively high in each grade, with primary school students and high school students having higher levels of home-school cooperation than middle school students. There were significant differences (*p* < 0.001) in school bullying, depression, academic anxiety, and home-school cooperation among primary and secondary school students in different provinces. The degree of school bullying among primary and secondary school students in six provinces was relatively low, with Guangdong and Hunan slightly higher than the other four provinces. The proportion of depression scores among primary and secondary school students in various provinces was almost similar, with a large proportion of low depression scores among primary and secondary school students in Shandong Province. The proportion of scores for primary and secondary school students in six provinces was also similar and relatively high. The proportion of home-school cooperation scores for primary and secondary school students in Hunan, Shandong, Sichuan, and Guangxi was higher than that in Hubei and Guangdong.

**Table 3:** Comparative analysis.

**Category**		**Academic anxiety (*M* ±*SD*)**	**Depression (*M* ±*SD*)**	**Home-school cooperation (*M* ±*SD*)**	**School bullying (*M* ± *SD*)**
Province	Hubei	8.08 ± 4.13	6.03 ± 5.54	4.76 ± 4.08	0.89 ± 2.10
	Hunan	8.14 ± 4.33	6.31 ± 5.61	7.03 ± 4.56	1.26 ± 2.60
	Shandong	6.49 ± 4.59	4.58 ± 5.24	8.58 ± 5.71	0.46 ± 1.61
	Guangdong	8.65 ± 3.86	7.62 ± 5.77	5.30 ± 3.81	1.44 ± 3.00
	Sichuan	7.51 ± 4.31	6.19 ± 5.13	7.32 ± 4.21	1.03 ± 2.38
	Guangxi	7.50 ± 3.87	5.42 ± 5.40	7.60 ± 4.81	0.75 ± 1.83
	*F*	20.27^***^	22.39^***^	61.85^***^	13.79^***^
Gender	Females	8.24 ± 7.27	6.67 ± 5.57	6.85 ± 6.49	0.85 ± 2.17
	Males	7.27 ±−6.66	5.57 ±−5.73	6.49 ±−2.20	1.14 ± 2.56
	*t*	−6.66^***^	−5.73^***^	−2.20^*^	3.50^***^
Grade	Second grade	6.00 ± 0.00	0.00 ± 0.00	0.00 ± 0.00	0.00 ± 0.00
	Third grade	2.38 ± 3.40	2.81 ± 4.25	9.00 ± 3.24	0.00 ± 0.00
	Fourth grade	3.40 ± 3.25	3.40 ± 4.21	11.21 ± 4.42	0.48 ± 1.71
	Fifth grade	6.29 ± 4.64	4.10 ± 4.65	8.31 ± 5.02	1.13 ± 2.43
	Sixth grade	6.94 ± 4.40	5.04 ± 5.57	7.76 ± 4.76	1.31 ± 2.95
	Seventh grade	8.44 ± 4.04	5.75 ± 5.52	6.28 ± 4.16	0.90 ± 2.02
	Eighth grade	8.70 ± 4.04	7.25 ± 5.69	6.53 ± 4.64	1.22 ± 2.62
	Ninth grade	8.31 ± 3.86	7.01 ± 5.23	8.86 ± 5.11	0.69 ± 1.80
	Tenth grade	8.39 ± 3.84	7.44 ± 5.75	3.88 ± 3.53	0.61 ± 1.89
	Eleventh grade	8.29 ± 3.69	7.57 ± 5.12	5.70 ± 4.09	0.95 ± 2.43
	Twelfth grade	10.00 ± 3.30	7.00 ± 3.94	6.70 ± 3.37	1.30 ± 2.16
	*F*	27.18^***^	21.92^***^	45.88^***^	4.19^***^

^***^p < 0.001, ^*^p < 0.05.

### 3.3 Correlation analyses

Table 4 depicts correlations between school bullying, depression, academic anxiety, and home-school cooperation. School bullying, depression, and academic anxiety were all negatively correlated with home-school cooperation; School bullying and academic anxiety were positively associated with depression; School bullying and academic anxiety was positively related.

**Table 4:** Pearson's correlations.

**Variable**	**Mean value**	**Standard deviation**	**1**	**2**	**3**	**4**
Home-school cooperation	6.67	4.74	1			
School bullying	1.00	2.38	−0.05^**^	1		
Depression	6.12	5.55	−0.21^**^	0.22^**^	1	
Academic anxiety	7.75	4.27	−0.23^**^	0.16^**^	0.40^**^	1

^**^p < 0.01.

### 3.4 Common method bias test

This study used Harman's single factor test to perform a common method bias test on all variables (Harman, 1960). The results showed that there were 5 factors with eigenvalues >1, among which the explanatory variation of the first common factor was 24%, which was less than the critical value of 40%, indicating that there was no significant common method bias in the data of this study.

### 3.5 Mediating effect test

The SPSS PROCESS macro (model 4) proposed by Hayes (2013) was used to test the mediating effect. We used gender, province, and grade as control variables, school bullying as the independent variable, depression as the dependent variable, and academic anxiety as the mediating variable to establish a mediation model. The results are shown in Tables 5, 6. School bullying was a significant predictor of depression (β = 0.24, *t* = 14.92, *p* < 0.001). When examining the mediating effect of academic anxiety, the ability of school bullying to predict depression remained significant (β = 0.19, *t* = 11.93, *p* < 0.001). Academic anxiety also had a significant positive predictive effect on depression (β = 0.33, *t* = 20.46, *p* < 0.001). The Bootstrap 95% confidence intervals for the direct effect of school bullying on depression and the mediating effect of academic anxiety did not contain 0, indicating that school bullying not only directly predicted depression, but also predicted depression through the mediating effect of academic anxiety. The direct effect (0.4) accounts for 77% of the total effect, while the mediating effect (0.1) accounts for 23% of the total effect.

**Table 5:** Mediating effect of academic anxiety.

**Variable**	**Depression**		**Depression**		**Academic anxiety**	
	β	***t***	β	***t***	β	***t***
School bullying	0.19	11.93^***^	0.24	14.92^***^	0.17	10.46^***^
Gender	−0.08	−5.00^***^	−0.12	−7.40^***^	−0.13	−7.93^***^
Province	0.03	1.66	0.02	1.15	−0.02	−1.23
Grade	0.18	11.15^***^	0.25	15.17^***^	0.22	13.16^***^
Academic anxiety	0.33	20.46^***^				
*R* ^2^	0.22		0.12		0.09	
*F*	189.67		117.66		79.80	

^***^p < 0.001.

**Table 6:** Mediating effects, direct effects, and total effects.

**Effects**	**Effect**	**BootSE**	**BootLLCI**	**BootULCI**	**Proportion of effect**
Mediating effects	0.13	0.02	0.10	0.16	0.23
Direct effects	0.43	0.04	0.35	0.52	0.77
Total effects	0.57	0.05	0.48	0.66	

### 3.6 Moderating effect test

**Figure 2:**
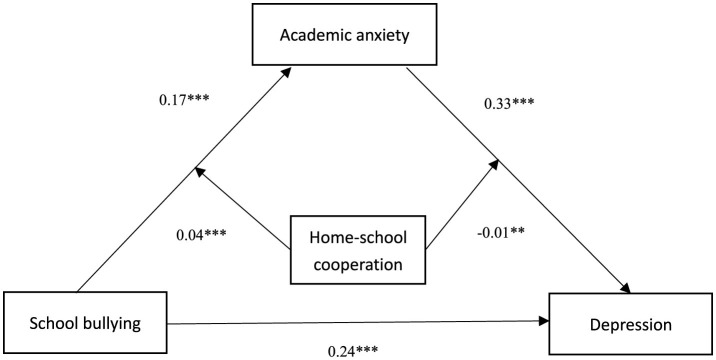
The path coefficients in the moderated mediation model. ***p < 0.001, **p < 0.01.

The SPSS PROCESS macro (model 58) proposed by Hayes (2013) was used to test the moderating effect. This study examined the moderating effect of home-school cooperation, with gender, province, and grade as control variables, school bullying as independent variable, depression as dependent variable, and academic anxiety as mediating variable. After incorporating home-school cooperation into the model, the results were shown in Tables 7, 8, the path coefficients in the moderated mediation model was shown in Figure 2. The interaction term between school bullying and home-school cooperation had a significant predictive effect on academic anxiety (academic anxiety: *B* = 0.04, *t* = 5.46, *p* < 0.001), indicating that home-school cooperation could regulate the predictive effect of school bullying on academic anxiety. The interaction term between academic anxiety and home school cooperation has a significant predictive effect on depression (depression: *B* = −0.01, *t* = −3.32, *p* < 0.01), which indicated that home-school cooperation were able to regulate the predictive effect of academic anxiety on depression.

**Table 7:** Testing for the moderated mediation effect.

**Outcome variable**	**Predictor variable**	***R***	***R* ^2^**	***F***	***B***	***SE***	***t***
Academic anxiety		0.35	0.13	79.65			
	Province				−0.00	0.05	−0.07
	Grade				0.35	0.04	9.78^***^
	Gender				−1.13	0.14	−8.16^***^
	School bullying				0.05	0.05	0.89
	Home-school cooperation				−0.19	0.02	−11.94^***^
	School bullying × home-school cooperation				0.04	0.01	5.46^***^
		0.48	0.23	142.91			
Depression	Province				0.12	0.06	2.24^*^
	Grade				0.42	0.04	9.72^***^
	Gender				−0.90	0.17	−5.27^***^
	School bullying				0.43	0.04	11.99^***^
	Academic anxiety				0.50	0.04	14.09^***^
	Home-school cooperation				−0.01	0.03	−0.42
	Academic anxiety × home-school cooperation				−0.01	0.00	−3.32^**^

^***^p < 0.001, ^**^p < 0.01, ^*^p < 0.05.

**Table 8:** Mediating effects of school cooperation at different levels.

**Path**	**Home-school cooperation**	**Effect**	***SE***	**LLCI**	**ULCI**
The first half of the intermediary path	(*M* – 1 *SD*) 1.93	0.12	0.04	0.04	0.21
	(*M*) 6.67	0.31	0.03	0.25	0.37
	(*M* + 1 *SD*) 11.41	0.50	0.09	0.40	0.59
The second half of the intermediary path	(*M* – 1 *SD*) 1.93	0.47	0.03	0.42	0.53
	(*M*) 6.67	0.41	0.02	0.37	0.45
	(*M* + 1 *SD*) 11.41	0.35	0.03	0.30	0.40

**Figure 3:**
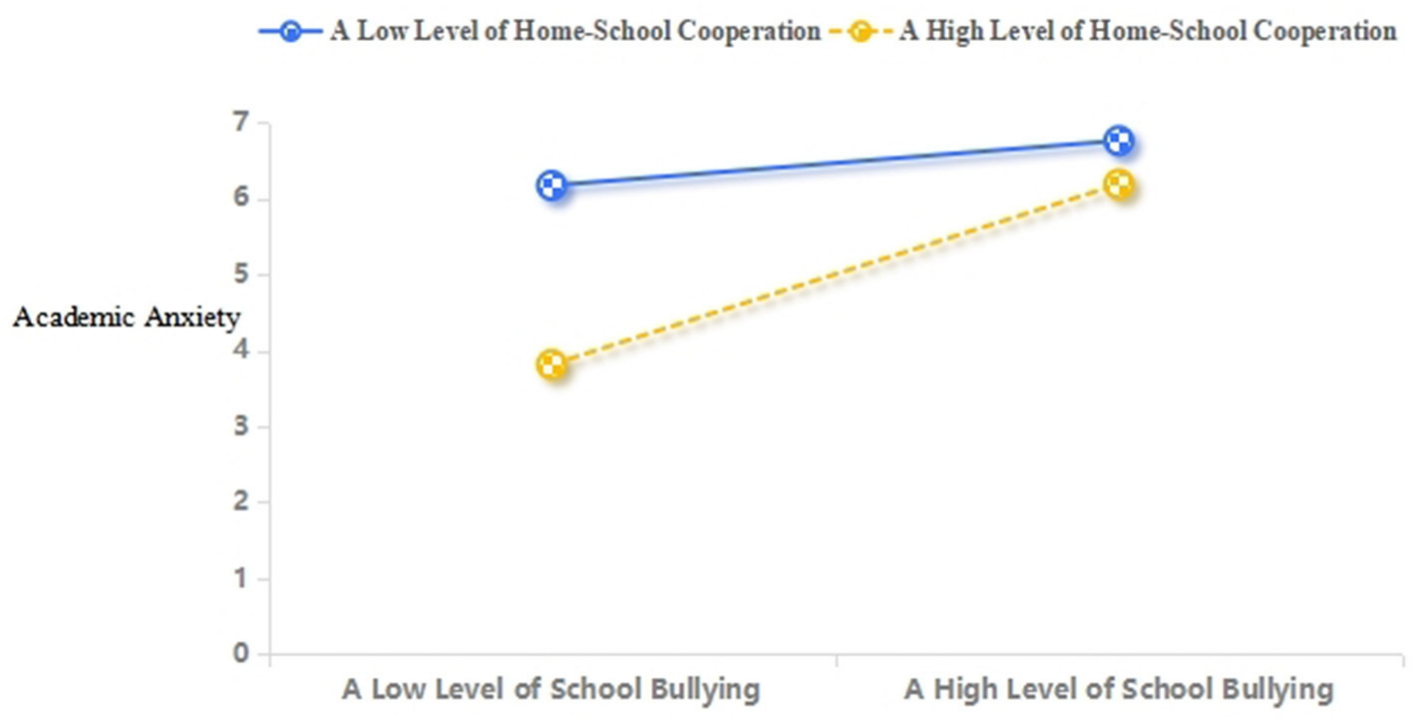
Home-school cooperation moderates the relation between school bullying and academic anxiety.

**Figure 4:**
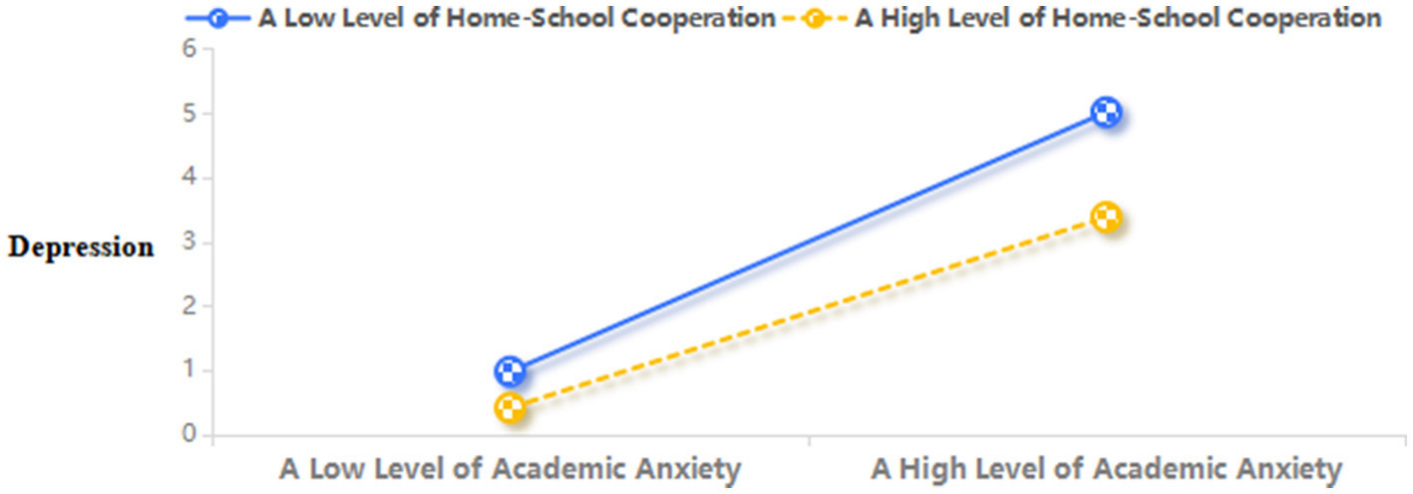
Home-school cooperation moderates the relation between academic anxiety and depression.

Furthermore, this study conducted further simple slope analysis. As shown in Figure 3, the degree of home-school cooperation was relatively high (*M* + 1 *SD*), and school bullying had a significant positive predictive effect on academic anxiety (simple slope = 0.62, *t* = 9.29, *p* < 0.001). The degree of home-school cooperation was relatively low (*M* – 1 *SD*), and school bullying still positively predicted academic anxiety (simple slope = 0.15, *t* = 4.00, *p* < 0.001), while the trend was weakening. With the increase of school bullying, the role of home-school cooperation weakened, and the intervention effect of home-school cooperation on depression was stronger when the degree of school bullying was low. From Figure 4, it can be seen that the degree of home-school cooperation was low (*M* – 1 *SD*), and academic anxiety significantly positively predicted depression (simple slope = 0.50, *t* = 14.09, *p* < 0.001). The degree of home-school cooperation was relatively high (*M* + 1 *SD*), and academic anxiety also significantly positively predicted depression (simple slope = 0.26, *t* = 5.46, *p* < 0.001), but its predictive effect was relatively small. This indicated that as the degree of home-school cooperation increased, the predictive effect of academic anxiety on depression gradually decreased.

## 4 Discussion

The aim of this study was to examine the effects of school bullying on depression in Chinese primary and secondary school students after the COVID-19 and the effects of academic anxiety and home-school cooperation on the relationship between school bullying and depression. We found that school bullying is a significantly positive predictor of depression, and academic anxiety has a mediating effect on the relationship between school bullying and depression. More importantly, home-school cooperation has moderating effects on the relationship between school bullying and academic anxiety and the relationship between academic anxiety and depression.

### 4.1 Effects of school bullying on depression

The results of the study showed that school bullying is a positive predictor of depression in Chinese primary and secondary school students after the COVID-19, implying that school bullying is a significant risk factor for depression among primary and secondary school students, which is consistent with previous research findings (Troop and Ladd, 2005; Chen et al., 2024). Primary and secondary school students who suffer from school bullying are often vulnerable to serious damage to their individual self-esteem, and they tend to internalize negative information from bullies, leading to depression. Primary and secondary school students who are subjected to school bullying may be marginalized in their peer group, gradually withdrawing from mainstream society, manifested as panic toward the group, peer rejection, and thus easily developing depression in loneliness (Xu et al., 2024). In addition, primary and secondary school students who suffer from school bullying may make biased attributions, attributing their depression to self blame for bullying from their peers in school (Liu and Lu, 2017). This result provides strong evidence for that school bullying is a positive predictor of depression in Chinese primary and secondary school students after the COVID-19 and supports hypothesis 1.

### 4.2 Mediating effect

The results of mediating effect test shows that academic anxiety among primary and secondary school students is not only positively correlated with depressive emotions, but also plays a significant mediating role between school bullying and depressive emotions, which are consistent with previous related research results (Zhang and Chen, 2016). Primary and secondary school students who suffer from school bullying may feel helpless in school, lose confidence in their studies, and experience increasing academic anxiety if they choose to remain silent and lack external regulation. The more severe the academic anxiety felt by primary and secondary school students, the more likely they are to fall into depression. Bullying often occurs among students whose personalities are erased by heavy academic burdens, leading to feelings of inferiority and disappointment (Liu and Gong, 2009). Primary and secondary school students who suffer from school bullying may also feel physically and mentally exhausted, nervous, depressed, and even panic about the school, resulting in serious academic anxiety. School is an important source of academic anxiety and psychological pressure for primary and secondary school students. Bullying, interpersonal difficulties, and friction in school can make students feel uneasy and anxious about their studies, leading to heavy academic anxiety and triggering depression (Hu, 2018). Academic anxiety has a mediating effect on the relationship between school bullying and depression, supporting hypothesis 2.

### 4.3 Moderating effect

The moderation effect analysis shows that the effect of school bullying on academic anxiety is moderated by home-school cooperation, and the effect of academic anxiety on depression is also moderated by home-school cooperation, which are consistent with the conclusions of existing research (Li and Gu, 2023). The interaction term between school bullying and home-school cooperation has a significant positive predictive effect on academic anxiety. Home-school cooperation can moderate the relationship between school bullying, academic anxiety, and depression, while high level home-school cooperation can buffer the impact of school bullying on academic anxiety. The families of students who engage in school bullying, as well as the families of bullied students, are characterized by a lack of emotional warmth and democratic atmosphere. Parents often educate their children through rough punishment, and conflicts and violence are prevalent between parents (Stevens et al., 2002), resulting in a lack of high-level cooperation between families and schools. Parents lack the emotional warmth needed for their children's mental health development, as well as their incompetence in teaching their children basic survival skills, making primary and secondary school students more likely to be bullied (Su et al., 2016). Therefore, schools need to actively strengthen home-school cooperation through home visits, parent-teacher meeting, online schools for parents, and other means (Yao, 2017). By carrying out high-level home-school cooperation, the adverse effects of academic anxiety caused by school bullying on primary and secondary school students can be effectively reduced. In order to help primary and secondary school students who have been bullied, we should not rely solely on home-school cooperation, but should take multiple measures to carry out collaborative strategies among family, school, and society.

The interaction term between academic anxiety and home-school cooperation has a significant negative predictive effect on depression. Compared with primary and secondary school students with high level home-school cooperation, academic anxiety is more likely to cause depression in primary and secondary school students with low level home school cooperation. Due to academic anxiety, primary and secondary school students with low level home-school cooperation are not only prone to academic anxiety, but also prone to depression, while primary and secondary school students with high level home-school cooperation are less likely to experience academic anxiety and depression. When the emotional regulation ability of primary and secondary school students is impaired, emotional anxiety will increase, which usually exacerbates the risk of anxiety and depression (Wang et al., 2014). Primary and secondary school students who receive good emotional support at home and school generally have better emotional adaptation, are better able to adapt to academic tasks, and are less prone to depression. Lacking support from family and school is one of the risk factors for students to experience academic anxiety. Depression is correlated with uncontrollable academic anxiety, and support from parents and peers can help primary and secondary school students cope with and alleviate academic anxiety, thereby reducing depression. It is worth noting that the support from family and school has a mutually reinforcing effect on depression, and neither support can compensate for each other's negative impact on reducing depression (Tian et al., 2012). In other words, the joint support of families and schools is more helpful in alleviating the depression caused by academic anxiety for primary and secondary school students. Home-school cooperation has moderating effects on the relationship between school bullying and academic anxiety and the relationship between academic anxiety and depression. This supports hypothesis 3.

### 4.4 Limitations and applications

Even though this study provided valuable findings on how school bullying affects depression and academic anxiety. This study still has certain limitations: it is a cross-sectional study and cannot determine causality and individual developmental differences between school bullying, depression, academic anxiety and home-school cooperation, and other related behaviors. In the future, longitudinal studies should be conducted to explore their deeper associations. Therefore, a longitudinal design can be used in future studies to better validate the moderated mediation model and examine the causal relationships between school bullying, depression, academic anxiety and home-school cooperation.

Despite its limitations, this study has significant theoretical and practical significance. Firstly, this research is beneficial for expanding the research field on the relationship between school bullying, depression, academic anxiety, and home-school cooperation among Chinese primary and secondary school students under the background of China's “Double Reduction” education policy. Exploring the mediating role of academic anxiety and the moderation effect of home-school cooperation, this study provides detailed insights into how families and schools can prevent and treat depression for primary and secondary school students. Secondly, this research established a moderated mediation model based on existing relevant research to explore the impact of school bullying on depression among Chinese primary and secondary school students, analyzing the mediating role of academic anxiety and the moderating role of home-school cooperation. The research results indicate that school bullying is positively correlated with depression among Chinese primary and secondary school students. Academic anxiety mediates the relationship between school bullying and depression. Home-school cooperation plays a moderating role in the first and second half of the mediating effect. High level home-school cooperation can buffer the impact of school bullying on academic anxiety. Compared with primary and secondary school students with high level home-school, academic anxiety is more likely to cause depression among primary and secondary school students with low level home-school. Thirdly, this research can provide theoretical support and reference for effectively resolving school bullying and preventing and intervening in depression among primary and secondary school students. At the same time, the results of this study imply that in order to prevent and respond to school bullying, it is necessary to leverage and improve the roles of schools, families, and governments (Wei and Fan, 2016). With the government as the lead, family counseling services should be provided to increase teachers and students' awareness of school bullying, and a school bullying prevention and control plan should be systematically constructed (Hu, 2017). By improving the admission assessment system and coordinating with families, schools, and communities to continuously manage extracurricular training, we can truly alleviate the academic anxiety of primary and secondary school students. Strengthening mental health education for primary and secondary school students is urgent. Preventing and intervening in depression among primary and secondary school students can be achieved through strengthening cooperation between families and schools. Families and schools need to strengthen primary and secondary school students' psychological quality training, enhancing the effectiveness of preventing and treating psychological problems among primary and secondary school students (Feng and Zhang, 2005).

## 5 Conclusion

This study found that school bullying is a significant positive predictor of depression in Chinese primary and secondary school students after the COVID-19, and academic anxiety has a mediating effect on the relationship between school bullying and depression. In addition, home-school cooperation has moderating effects on the relationship between school bullying and depression and the relationship between academic anxiety and depression. Moderate home-school cooperation can help reduce the impact of school bullying on academic anxiety and depression, as well as the impact of academic anxiety on depression. The moderation and mediation model in this study are able to help to better understand the correlation between school bullying and depression in Chinese primary and secondary school students after the COVID-19.

## Data Availability

The original contributions presented in the study are included in the article/Supplementary material, further inquiries can be directed to the corresponding author.
